# Interaction capacity as a potential driver of community diversity

**DOI:** 10.1098/rspb.2021.2690

**Published:** 2022-02-23

**Authors:** Masayuki Ushio

**Affiliations:** ^1^ Hakubi Center, Kyoto University, Kyoto 606-8501, Japan; ^2^ Center for Ecological Research, Kyoto University, Otsu 520-2113, Japan

**Keywords:** community diversity, empirical dynamic modelling, environmental DNA, interaction network, quantitative metabarcoding

## Abstract

How patterns in community diversity emerge is a long-standing question in ecology. Studies suggested that community diversity and interspecific interactions are interdependent. However, evidence from high-diversity ecological communities is lacking because of practical challenges in characterizing speciose communities and their interactions. Here, I analysed time-varying interaction networks that were reconstructed using 1197 species, DNA-based ecological time series taken from experimental rice plots and empirical dynamic modelling, and introduced ‘interaction capacity', namely, the sum of interaction strength that a single species gives and receives, as a potential driver of community diversity. As community diversity increases, the number of interactions increases exponentially but the mean interaction capacity of a community becomes saturated, weakening interspecific interactions. These patterns are modelled with simple mathematical equations, based on which I propose the ‘interaction capacity hypothesis': that interaction capacity and network connectance can be two fundamental properties that influence community diversity. Furthermore, I show that total DNA abundance and temperature influence interaction capacity and connectance nonlinearly, explaining a large proportion of diversity patterns observed in various systems. The interaction capacity hypothesis enables mechanistic explanations of community diversity. Therefore, analysing ecological community data from the viewpoint of interaction capacity would provide new insight into community diversity.

## 1. Introduction

How patterns in community diversity in nature emerge is one of the most challenging and long-standing questions in ecology [1]. Community diversity, or species diversity, a surrogate of biodiversity that is most commonly focused on [2], is a collective consequence of community assembly [3]. In the community assembly processes, interspecific interactions, which contribute to the process of selection, play an important role in shaping community diversity particularly at a local (i.e. small/short spatio-temporal) scale [4,5]. They have played a central role when devising theories of ecological communities, including modern coexistence theory [6], niche theory [7] and many others. Understanding how interspecific interactions shape community diversity is key to understanding how patterns in an ecological community emerge in nature.

Theoretical studies and simple manipulative experiments have supported the view that interspecific interactions contribute to community diversity [4,8–13]. Nonlinear, state-dependent interspecific interactions have been shown to influence community diversity, composition and even dynamics [8,9,12], and weak interactions are key to the maintenance of community diversity [10,14]. However, despite enormous efforts to understand the interdependence between interspecific interactions and community diversity, especially efforts made in theoretical studies (e.g. [9,11–13]), whether and how interspecific interactions control diversity in a complex, high-diversity, empirical ecological community remain poorly understood. This is largely due to two difficulties: (i) the number of species and interspecific interactions examined in previous theoretical and experimental studies have been limited compared with those of a real, high-diversity ecological community under field conditions, and (ii) detecting causal relationships between interspecific interactions and diversity is not straightforward because manipulative experiments, a most effective strategy to detect causality, are not feasible when a large number of species and interactions are targeted under field conditions. Nonetheless, understanding the mechanism by which interspecific interactions drive community diversity in nature is necessary for predicting responses of ecological communities and their functions to the ongoing global climatic and anthropogenic threats [15].

To overcome the previous limitations and examine the causal relationships between interspecific interactions and diversity of a speciose, empirical community, I integrated quantitative environmental DNA monitoring and a nonlinear time-series analysis and found the potential influences of the capacity of species for interspecific interactions on community diversity. Efficient water sampling, DNA extraction and quantitative MiSeq sequencing with an internal standard DNA [16,17] overcame the first difficulty noted above: quantitative, highly diverse, multi-taxonomic, daily, 122-day-long ecological time series were obtained from five experimental rice plots under field conditions. This extensive ecological time series was analysed using a framework of nonlinear time series analysis, empirical dynamic modelling (EDM) [18–20], to overcome the second difficulty: EDM quantified fluctuating interaction strengths, reconstructed the time-varying interaction network of the ecological communities, and detected potential causal relationships between network properties and community diversity. Here, I look specifically at how interaction strengths change with community diversity, and how interspecific interactions and community diversity are causally coupled. Then, I derive a hypothesis that could explain community diversity in various empirical systems, which I call the ‘interaction capacity hypothesis'.

## 2. Results and discussion

### (a) Experimental design and ecological community monitoring

**Figure 1.**
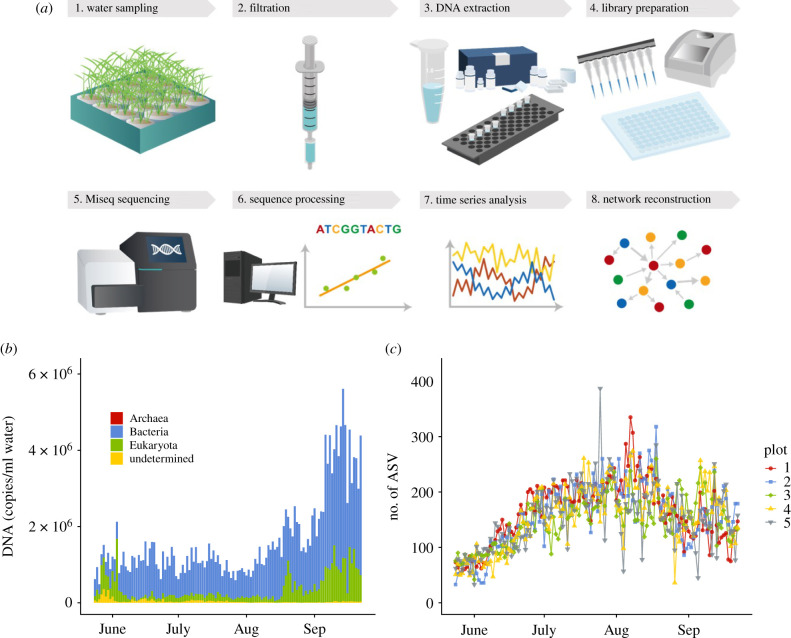
Workflow of the present study and time series of the rice plot ecological communities. (*a*) Workflow of the present study. (*b*) Mean DNA copy number of the ecological communities in rice plots. Different colours indicate different superkingdoms. (*c*) Temporal patterns of the number of ASVs detected from each plot. Different symbols and colours indicate different rice plots (*n* = 122 for each plot; total *n* = 610).

Ecological time series were taken from five experimental rice plots established at the Center for Ecological Research, Kyoto University, Japan (figure 1*a*; electronic supplementary material, figure S1). Ecological communities were monitored by analysing DNA in water samples taken from the rice plots using two types of filter cartridge [16]. Daily monitoring during the rice-growing season of 2017 (23 May to 22 September) resulted in 1220 water samples in total (5 plots × 2 filter types [*φ*0.45 µm and *φ*0.22 µm filters] × 122 days). Prokaryotes and eukaryotes (including fungi and animals) were analysed by amplifying and sequencing 16S rRNA, 18S rRNA, ITS (for which DNAs extracted from *φ*0.22 µm filters were used) and mitochondrial COI regions (for which DNAs from *φ*0.45 µm filters were used), respectively, using quantitative MiSeq sequencing [16,17]. Over 80 million reads were generated by four runs of MiSeq, and the sequences generated were then analysed using the amplicon sequence variants (ASVs) method [21] (see electronic supplementary material, figure S2 for the sequence quality). Examination of the relationships between the copy numbers and sequence reads of standard DNAs (electronic supplementary material, figure S3*a*,*b*) and comparisons of the quantitative MiSeq method with three independent analyses showed that the quantitative MiSeq sequencing has a reasonable capability to measure the quantity, composition and diversity of the ecological communities (electronic supplementary material, figure S3*c*–*i*). Among over 10 000 ASVs detected, 1197 ASVs (equivalent to 1197 taxonomic units) were abundant, frequently detected and contained enough temporal information for subsequent time-series analyses (see the electronic supplementary material, Methods).

### (b) Community dynamics and reconstruction of fluctuating interaction network

The total DNA copy number increased late in the sampling period (figure 1*b*). By contrast, ASV diversity (a surrogate of species diversity in the present study) was highest in August and then decreased in September (figure 1*c*). Prokaryotes largely accounted for this pattern (electronic supplementary material, figure S4), which is not surprising given their higher diversity and abundance compared with the other taxa.

Fluctuating interaction networks were reconstructed using EDM, a time-series analytical framework for nonlinear dynamics [18–20]. In the analysis, I detected interacting ASV pairs using convergent cross-mapping (CCM) [18], a causality test of EDM and then quantified the interaction strengths by multivariate, regularized S-maps [20,23,24] (a locally weighted linear regression tool of EDM). Linear trends of air temperature during the monitoring season were included in the S-maps, and thus the interaction strength estimated here reflect net interactions between species (see the electronic supplementary material, Methods). In addition, although the total number of ASVs analysed was over 1000, most ASVs have fewer than 20 causal interactions (electronic supplementary material, figure S5a), suggesting that the estimation of interaction strengths by S-map would be reliable (i.e. the number of data points is more than or roughly equal to the square of the dimensions of reconstructed state space, which is required for robust estimations of the S-map coefficients). Note that, in the case that the number of dimensions far exceeds the number of data points, a recently proposed S-map method would be more suitable [25].

**Figure 2.**
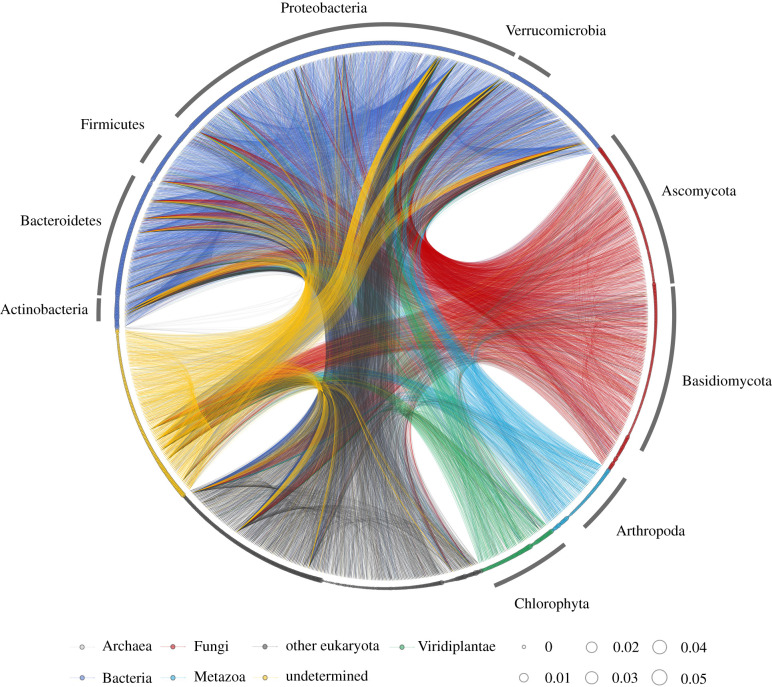
Reconstructed interaction network of the ecological community. Lines indicate causal influences between nodes, and line colours indicate causal taxa (e.g. blue lines indicate the causal influences from a bacterial ASV to another ASV). The relative size of each node (circle) represents the total DNA copy number of the ASV (*n* = 1197 for nodes). Different colours of nodes indicate different taxa, as shown at the bottom. Note that, although interaction strengths were quantified at each time point, the information on the time-varying interactions are not shown in the network. The detailed, daily fluctuating interaction network is presented in electronic supplementary material, figure S5b and at https://doi.org/10.6084/m9.figshare.16456179 as an animation.

Figure 2 shows the reconstructed network of the detected interactions over the monitoring period (for the time-varying interaction networks, see electronic supplementary material, figure S5*b* and the electronic supplementary material, video; https://doi.org/10.6084/m9.figshare.16456179). The properties of the ecological network changed over time. For example, relatively dense interactions among community members in July and August (in Plot 1) disappeared by September (electronic supplementary material, figure S5*b*). Interestingly, dynamic stability [26], an index that quantifies how fast the community bounces back from small perturbations (i.e. the dominant eigenvalue of the interaction matrix), was almost always over 1, suggesting unstable community dynamics (electronic supplementary material, figure S5*c*–*g*). This pattern may not be surprising because the rice plots were open systems under field conditions. Many community members could immigrate and emigrate, leading to inherently unstable community dynamics. Analysis of the dynamic stability of subset communities suggested that fluctuations in moderately abundant community members could contribute to the unstable dynamics (electronic supplementary material, figure S5*d*–*g*). The fluctuating, seemingly unstable conditions might be key to understanding the coexistence of many community members in nature. Alternatively, calculating and comparing different stability measures such as structural stability [27] may provide a different insight.

### (c) Patterns emerging in the interaction networks

**Figure 3.**
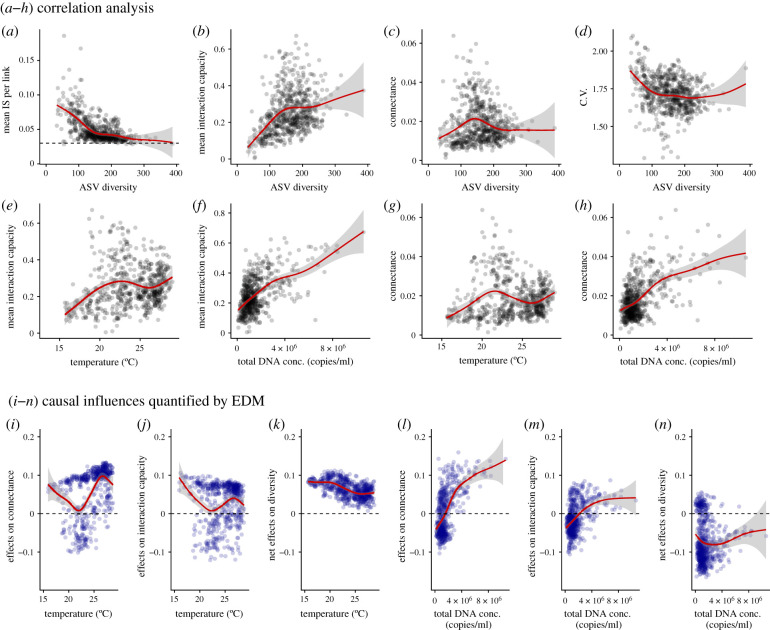
Relationships between the interaction network properties. (*a*–*d*) Covarying relationships (correlations) between ASV diversity and properties of the interaction network, namely the mean interaction strength (*a*), interaction capacity (*b*), connectance (*c*) and coefficients of variations in population dynamics (*d*). Interaction capacity is defined as the sum of absolute values of interaction strength that a species gives or receives. Dashed line in a indicates a converged value of mean interaction strength (approx. equal to 0.03). (*e*–*h*) Relationships between interaction capacity, connectance, mean air temperature and total DNA copy numbers. (*i*–*n*) Causal influences of air temperature and total DNA copy numbers on connectance, interaction capacity and community diversity quantified by EDM. CCM was first applied to each pair, and then multivariate, regularized S-map was applied to quantify the causal influences. Red lines indicate statistically significant nonlinear regressions by general additive model and grey shaded region indicate 95% confidence interval.

Properties of the interaction networks showed intriguing patterns (figure 3; electronic supplementary material, table S1). As ASV diversity increases, the mean interaction strength per link decreases (figure 3*a*; for mathematical definitions of network properties, see the electronic supplementary material, Methods), while the number of interactions in a community increases exponentially (electronic supplementary material, figure S6), being consistent with the theoretical and experimental evidence [4,10]. This suggests that the total interaction strength that a species receives and gives, which I call ‘interaction capacity’, might not exceed a certain upper limit, even when ASV diversity and the number of interactions in a community increase. Note that ‘interaction capacity’ should ideally be defined as an index of the total amount of available energy, resources and time that can be invested in interspecific interactions. However, in the present study, the interaction capacity is measured as the total interaction strength, which is actually ‘realized’ interaction capacity. In reality, the availability of energy, resources and time, which are required to interact with other species, is limited. For example, it seems difficult for a single species to strongly interact with a large number of species within a certain time interval given their constrained body size, abundance and generation time. Also, interspecific interactions usually involve the consumption of a certain amount of energy. Thus, it is intuitively plausible to assume that there is a certain upper limit of interaction capacity. Indeed, the upward trend of the mean ‘realized’ interaction capacity is weakened when ASV diversity is over 100 in the studied system (figure 3*b*), supporting the assumption.

Another important property of the interaction networks, connectance, is also relatively constant as ASV diversity varies (figure 3*c*; for the definition of connectance, see the electronic supplementary material, Methods). Importantly, these patterns were not reproduced when the randomly shuffled version of the original time series was analysed (electronic supplementary material, figure S7). In addition, this pattern of interaction strength per link decreasing and converging when the number of interactions and/or species is high and the relatively constant connectance are valid even at the species level (electronic supplementary material, figure S8). These findings suggest that the original results may not have been experimental or statistical artefacts but rather may have emerged as consequences of empirical community assembly processes. Another intriguing pattern is that mean values of coefficient of variation (CV) of DNA copy numbers, an EDM-independent index of realized temporal variability, decrease as a function of ASV diversity (figure 3*d*; one outlier shows a relatively high CV regardless of a high community diversity). This showed, for the first time, that the small temporal variability in species abundances observed in plant communities [28] is also valid even when all major cellular organisms are taken into account, and that there is a connection between community diversity and temporal dynamics in this system.

### (d) Interdependence of community diversity and interaction capacity revealed by simple mathematical equations

To better understand and clarify the implications of the patterns that emerged in the network properties, I explicitly show the relationship between the network properties by developing a simple mathematical model. By starting with minimal assumptions, I demonstrate that community diversity and interaction capacity are interdependent on each other. Since connectance, *C*, is defined as $C=N_{\mathrm{link}}/S^{2}$, species diversity (species richness), *S*, can be simply represented as $S=\sqrt{N_{\mathrm{link}}/C}$, where *N*_link_ indicates the total number of interactions in a community. Furthermore, *N*_link_ can be decomposed into the mean realized interaction capacity at the community level (*IC*), defined as $IC\;=2\times \sum_{\;j=1}^{S}\sum_{{}_{i\neq j}^{i=1}}^{S}|IS_{i\to j}|/S$ (see the electronic supplementary material, Methods), and the mean interaction strength per link, *IS*_link_, as follows:

2.1 $$\begin{array}{c} N_{\mathrm{link}}=\frac{IC}{2\times IS_{\mathrm{link}}}\times S. \end{array}$$

$IC/IS_{\mathrm{link}}$ is divided by 2 because each interaction strength is counted twice (for donor and receiver species). Therefore, *S*, *C*, *IC* and *IS*_link_ should satisfy the following relationship:

2.2 $$\begin{array}{c} S=\frac{IC}{2\times IS_{\mathrm{link}}\times C}\;. \end{array}$$

No system-specific assumption is used to derive equation (2.2), and thus *S*, *IC*, *IS*_link_ and *C* should satisfy equation (2.2) in any system and under any condition. Note that the four parameters are all interdependent, and thus a change in one parameter influences other parameters. Results of CCM of the time series taken from the rice plots suggested that the four parameters in equation (2.2) are indeed interdependent in the empirical communities (electronic supplementary material, figure S9).

The simple equation, equation (2.2), suggests that there is a negative relationship between *S* and *C* or *IS*_link_ given that the other parameters are constant. Similar arguments were made in previous seminal theoretical studies [11,13], but there are important contributions of equation (2.2) to understanding empirical ecological communities. ‘Interaction capacity’ is an easily understandable concept, and it can be defined as ‘trait’ for any biological level (e.g. for community, species or even local populations). Equation (2.2) explicitly shows the linkage between the interaction capacity and other fundamental properties of an ecological community, opening up a new research direction to link the biological trait and network properties. Furthermore, it would enable intuitive and clear explanations of mechanisms of community diversity by investigating how *IC* and *C* are determined and it has empirical supports as shown in the following sections.

### (e) The interaction capacity hypothesis

A potentially important feature of the emerging empirical patterns is that *IS*_link_ tends to converge when community diversity increases (figure 3*a*). When using the system-specific parameter value of converged *IS*_link_ in the present study, maximum possible community diversity, *S*_max_, in the rice plots is approximated based on equation (2.2) as follows:

2.3 $$\begin{array}{c} S_{\max}\approx \frac{IC}{2\times 0.03\times C}.\; \end{array}$$

Equation (2.3) predicts that there is a positive relationship between the interaction capacity and community diversity while there is a negative relationship between connectance and community diversity, which is consistent with previous theoretical studies (e.g. [11,13]). Although equation (2.3) alone is not sufficient to understand which variable is an ultimate determinant of the system properties, this mathematical model indicates that species diversity can be predicted if we know how *IC* and *C* are determined in a community.

My ecological time series provides a unique opportunity to detect potential variables that influence interaction capacity (*IC*) and connectance (*C*) under field conditions. In the analysis, I focused on two fundamental variables that are statistically independent of the network properties: air temperature and total DNA copy number (an index of total abundance/biomass). Also, I focused on these because air temperature is an independent external driver of community dynamics and because total abundance could be an index of net ecosystem productivity or available energy in a system, which could be a potential driver of community diversity [29,30]. Correlation analysis shows that the interaction capacity is positively correlated with mean air temperature and total DNA copy number (figure 3*e*,*f*). Connectance is positively correlated with total DNA copy number and is weakly correlated with mean air temperature (figure 3*g*,*h*).

Causal relationships between the network properties and external forces were examined with CCM, and the results suggested that mean interaction capacity and connectance are causally influenced by mean air temperature and total DNA copy number (electronic supplementary material, figure S9). The S-map revealed that mean air temperature in general positively influenced interaction capacity and connectance, as indicated by mostly positive values along the gradient (figure 3*i*,*j*; values on the *y*-axis indicate how changes in temperature cause changes in interaction capacity or connectance). Temperature may influence many aspects of biological processes (e.g. physiological rates of individuals), and therefore the influences of air temperature on interaction capacity and connectance may arise from the increased activities of individuals. Although temperature effects on connectance and mean interaction capacity at the community level were comparable, the net effects of temperature on diversity, that is, effects of temperature through its effects on interaction capacity and connectance, were consistently positive (figure 3*k*). This indicates that the positive influence of temperature on mean interaction capacity, not that on connectance, plays a major role in shaping community diversity.

When total DNA copy number is low, its influence on connectance is variable (figure 3*l*). When total DNA copy number is high, however, total DNA copy number strongly and positively influences connectance, suggesting that denser populations should have higher connectance, probably because the greater population size may facilitate random encounters among individuals or species. On the other hand, the influences on interaction capacity are relatively small and variable (figure 3*m*). These results predict that total DNA copy number (or abundance/biomass) over a certain threshold negatively influence diversity, which is indeed the case here (figure 3*n*).

Together, the results of EDM show that interaction capacity and connectance are influenced by temperature (*T*) and total DNA copy number (*DNA*). This suggests that, if we assume that *IS_link_* is converged in a system, species diversity, *S*, in the system can be approximated using these two fundamental parameters as follows:

2.4 $$\begin{array}{c} S\;\approx \frac{IC(T,DNA)}{2\times 0.03\times C(T,DNA)}\;. \end{array}$$

Although the influences of temperature and abundance (biomass or energy) on diversity have long been recognized in literatures (e.g. [31]), this model, supported by empirical evidence, provides mechanistic explanations about how temperature and abundance control community diversity. The present study did not include other potentially important abiotic factors such as water pH and nutrient availability, but the mechanisms of the influence of such factors may also be understood by considering their effects on interaction capacity and connectance. Because community diversity, interaction capacity and connectance are interdependent in any system and under any condition according to equation (2.2), the influences of any biotic/abiotic factors on community diversity can be mechanistically explained and predicted if we can understand the interdependence among these factors, interaction capacity and connectance, a proposal which I call the ‘interaction capacity hypothesis’.

### (f) Empirical evidence supporting the interaction capacity hypothesis in other systems and predictions based on the hypothesis

The simple mathematical model and the analyses of the extensive ecological time series reported here suggest that community diversity, interaction capacity and connectance are interdependent, and that the long-recognized patterns that temperature and total species abundance influence community diversity could be understood by considering their influences on interaction capacity and connectance. In some cases, variable responses of community diversity to temperature and/or abundance might be observed because of the nonlinear influences of temperature and abundance on interaction capacity and connectance (figure 3*i*–*n*). Conversely, if the hypothesis is applicable to other systems, it should be possible to explain community diversity reasonably well by a nonlinear regression using temperature and total abundance. Indeed, a meta-analysis that compiled two global datasets and four local datasets collected in Japan showed that biodiversity is surprisingly well explained only by a nonlinear regression using temperature and abundance, suggesting that the interaction capacity hypothesis might be applicable to a wide range of taxa and ecosystems (electronic supplementary material, figure S10*a*–*f* and Text; see also electronic supplementary material, figure S10*g*,*h* for other evidence).

**Figure 4.**
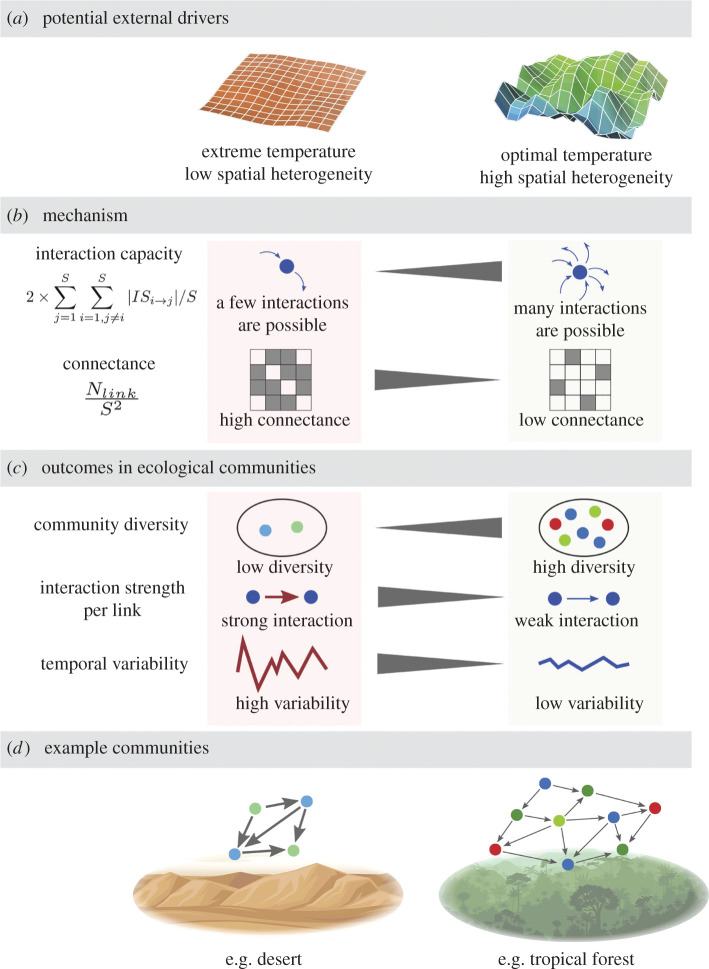
Potential drivers of the community network according to the interaction capacity hypothesis and examples of ecological communities. (*a*) Potential external drivers that contribute to the community diversity and network structure. (*b*) Mechanisms of community assembly. Extreme and optimal temperature would generally decrease and increase species interaction capacity, respectively. Spatial heterogeneity decreases connectance, which subsequently increases community diversity. (*c*) Outcomes of community diversity and dynamics. Extreme temperature and low spatial heterogeneity (e.g. desert ecosystem) generate a community with low diversity, high interaction strength (and a small number of interactions) and unstable dynamics. On the other hand, optimal temperature and high spatial heterogeneity (e.g. tropical forests) generate a community with high-diversity, low-interaction strength (but a large number of interactions) and stable community dynamics. (*d*) Examples of ecological communities (low-diversity versus high-diversity community) according to the potential external drivers and the interaction capacity hypothesis. (Online version in colour.)

The interaction capacity hypothesis provides quantitative and unique predictions about community diversity in nature. For example, everything else being equal, community diversity may increase with increasing temperature because of increased interaction capacity under warmer conditions (figure 4*a*,*b*). Similarly, community diversity will increase with increasing habitat heterogeneity because of decreased connectance (figure 4*a*,*b*). Thus, high-diversity communities will exist under optimal environmental conditions (i.e. high interaction capacity) with spatially heterogeneous habitats (i.e. low connectance), such as plants in tropical forests or microbes in soils with neutral pH (figure 4*c*,*d*). Furthermore, under such conditions, community dynamics will be stabilized because of the decreased interaction strengths (figures 3*d* and 4*c*; also see evidence from a recent experimental study [10]). On the other hand, low-diversity communities will exist under extreme environmental conditions with spatially homogeneous habitats, such as deserts, because of decreased (or consumed) interaction capacity and increased connectance.

### (g) Future directions

Although there are several potential limitations of the present study (see the electronic supplementary material for the details), the interaction capacity hypothesis has some empirical and theoretical supports, and thus how interaction capacity is determined will be an interesting question in ecology. For example, interaction capacity may be influenced by energy and resources provided to a system, but it can also be influenced by species identity (i.e. species' ecology, physiology and evolutional history; e.g. higher interaction capacities of prokaryotes than of eukaryotes; see electronic supplementary material, figure S8*b*,*d*). Also, rapid evolution and eco-evolutionary feedbacks may cause changes in species trait, their interactions and dynamics (e.g. [32]), which would influence how species assign their interaction capacity to biotic/abiotic interactions. Incorporating species identity, evolutionary history and eco-evolutionary feedbacks into the interaction capacity hypothesis would be an interesting direction for future studies. Furthermore, abiotic factors can easily and explicitly be incorporated into the interaction capacity hypothesis. For example, if interaction capacity (or energy resources) is ‘consumed’ to adapt to harsh environmental conditions, interaction capacity that can be used for interspecific interaction will decrease, which will consequently decrease community diversity. Incorporating the impact of climate change into the interaction capacity hypothesis might reveal implications for how ecological communities respond to climate change.

## 3. Conclusion

How patterns in community diversity emerge has been extensively studied by experimental and theoretical approaches, yet rarely examined for highly diverse, complex ecological communities. Using DNA-based, highly frequent, quantitative, extensive ecological time series and EDM, I proposed that ‘interaction capacity’ would be a key concept to understand and predict community diversity. Connectance may also play an important role, because it influences how interaction capacity is divided into each interaction link. Interaction capacity can be influenced by the total abundance and temperature, which can provide mechanistic explanations for many observed ecological patterns in nature. Carefully designed experiments (e.g. manipulation of interaction strength, capacity and/or community diversity) and large-scale monitoring of natural ecological communities are required to empirically verify the interaction capacity hypothesis. However, once validated, expanding spatial and temporal scales and incorporating the other processes in community assembly (that is, speciation, dispersal and drift) [3] into the interaction capacity hypothesis will further deepen our understanding of community assembly processes, which will contribute to how we can predict, manage and conserve biodiversity and the resultant ecosystem functions in nature.

## 4. Method summary

For the full method descriptions, see the electronic supplementary material, Methods.

### (a) Experimental setting

Five artificial rice plots were established using small plastic containers (90 × 90 × 34.5 cm; 216 l total volume; Risu Kogyo, Kagamigahara, Japan) in an experimental field at the Center for Ecological Research, Kyoto University, in Otsu, Japan (34̊ 58′ 18′′N, 135̊ 57′ 33′′E) (electronic supplementary material, figure S1). Sixteen Wagner pots (*φ*174.6 × *φ*160.4 × 197.5 mm; AsOne, Osaka, Japan) were filled with commercial soil, and three rice seedlings (var. Hinohikari) were planted in each pot on 23 May 2017 and then harvested on 22 September 2017 (122 days). The containers (hereafter, ‘plots’) were filled with well water, and the ecological community was monitored by analysing DNA in the well water (see following subsections).

### (b) Field monitoring of the ecological community

To monitor the ecological community, water samples were collected daily from the five rice plots. Approximately 200 ml of water in each rice plot was collected from each of the four corners of the plot using a 500 ml plastic bottle and taken to the laboratory within 30 min. The water was filtered using Sterivex filter cartridges (Merck Millipore, Darmstadt, Germany). Two types of filter cartridges were used to filter water samples: to detect microorganisms, *φ*0.22 µm Sterivex (SVGV010RS) filter cartridges that included zirconia beads inside were used [16], and to detect macroorganisms, *φ*0.45 µm Sterivex (SVHV010RS) filter cartridges were used. After filtration, 2 ml of RNAlater solution (ThermoFisher Scientific, Waltham, Massachusetts, USA) were added to each filter cartridge to prevent DNA degradation during storage. In total, 1220 water samples (122 days × 2 filter types × 5 plots) were collected during the census term. In addition, 30 field-level negative controls, 32 PCR-level negative controls with or without the internal standard DNAs and 10 positive controls to monitor the potential DNA cross-contamination and degradation during the sample storage, transport, DNA extraction and library preparations were used.

### (c) DNA extractions, library preparations for MiSeq sequencing with internal standard DNAs and sequence processing

Due to the space limitation, detailed information about how DNA was extracted, DNA library was prepared, and generated sequences were processed are described in the electronic supplementary material, Methods. Briefly, DNA was extracted and purified using a DNeasy Blood & Tissue kit following a protocol described in my previous study [16]. After the purification, DNA was eluted using 100 µl of the elution buffer and stored at −20°C until further processing.

Two-step PCR approach was adopted for the library preparation for quantitative MiSeq sequencing. Briefly, the first-round PCR (first PCR) was carried out with the internal standard DNAs to amplify metabarcoding regions using primers specific to prokaryotes (515F and 806R), eukaryotes (Euk_1391f and EukBr), fungi (ITS1-F-KYO1 and ITS2-KYO2) and animals (mlCOIintF and HCO2198) (for primer sequences and references, see the electronic supplementary material). The second-round PCR (second PCR) was carried out to append indices for different samples for sequencing with MiSeq. The DNA library was sequenced on the MiSeq (Illumina, San Diego, CA, USA).

Scripts to process the sequence data are available at Zenodo (https://doi.org/10.5281/zenodo.5867264). The raw MiSeq data were converted into FASTQ files using the bcl2fastq program provided by Illumina (bcl2fastq v.2.18). The FASTQ files were then demultiplexed using the command implemented in Claident v.0.2.2019.05.10 (http://www.claident.org) [33]. Demultiplexed FASTQ files were then analysed using the ASV method implemented in the DADA2 (v.1.11.5) package of R. Taxonomic identification was performed using Claident.

### (d) Estimations of DNA copy numbers and validation of the quantitative capability of the MiSeq sequencing with internal standard DNAs

For all analyses in this subsection, the free statistical environment R v.3.6.1 was used [34]. The procedure used to estimate DNA copy numbers consisted of two parts, following previous studies [16,17]. Briefly, I did (i) linear regression analysis to examine the relationship between sequence reads and the copy numbers of the internal standard DNAs for each sample (electronic supplementary material, figure S3*a*,*b*), and (ii) the conversion of sequence reads of non-standard DNAs to estimate the copy numbers using the result of the linear regression for each sample. The regression equation was MiSeq sequence reads = sample-specific regression slope × the number of standard DNA copies (per µl) (see electronic supplementary material, figure S3 for the validation of this method).

The quantitative capacity of the MiSeq sequencing with internal standard DNAs (i.e. the quantitative MiSeq sequencing) is one of important factors that could influence subsequent data analyses. Therefore, I checked the reliability of the quantitative capacity of the method using three independent experiments (fluorescent-based total DNA quantifications, quantitative PCR [qPCR] of the 16S region and shotgun metagenomic analysis) and compared the results with those of the quantitative MiSeq sequencing. Details of the total DNA quantification, qPCR and shotgun metagenomic analysis and discussion of the results are provided in the electronic supplementary material, Methods.

### (e) Empirical dynamic modelling: convergent cross-mapping and the regularized, multivariate S-map method

The reconstruction of the original dynamics using time-lagged coordinates is known as state space reconstruction (SSR) [35,36] and is useful when one wants to understand complex dynamics. Recently, developed tools for nonlinear time-series analysis called ‘empirical dynamic modelling (EDM),’ which were specifically designed to analyse state-dependent behaviour of dynamic systems, are rooted in SSR [18–20,26]. These methods do not assume any set of equations governing the system and thus are suitable for analysing complex systems, for which it is often difficult to make reasonable *a priori* assumptions about their underlying mechanisms.

To detect causation between species detected by the DNA analysis, CCM, a causality detection tool of EDM [18], implemented in ‘rEDM’ (v.0.7.5) [19,37] was performed with some modifications based on an algorithm described in ‘rUIC’ (v.0.1.5) packages [22]. After the detection of causal pairs, the multivariate, regularized S-map (sequential locally weighted global linear map) method was used to quantify dynamic (i.e. time varying) interactions of the causal pairs [20,24]. What criteria were used for each analysis, how statistical significance was determined, and other detailed information are described in the electronic supplementary material, Methods and https://doi.org/10.5281/zenodo.5867264. How properties of the interaction network were calculated after the network reconstruction are also described in the electronic supplementary material, Methods.

## Data accessibility

Computer codes used in the present study are available from Zenodo (https://doi.org/10.5281/zenodo.5867264) and GitHub (https://github.com/ong8181/interaction-capacity). Sequence data are deposited in DDBJ Sequence Read Archives (DRA) (DRA accession number = DRA009658, DRA009659, DRA009660 and DRA009661 for ecological community monitoring data and DRA012505 for supporting shotgun metagenomic data).

## References

[1] Gaston KJ. 2000 Global patterns in biodiversity. Nature **405**, 220-227. (10.1038/35012228) 10821282

[2] Willig MR, Kaufman DM, Stevens RD. 2003 Latitudinal gradients of biodiversity: pattern, process, scale, and synthesis. Annu. Rev. Ecol. Evol. Syst. **34**, 273-309. (10.1146/annurev.ecolsys.34.012103.144032)

[3] Vellend M. 2016 The theory of ecological communities. Princeton, NJ: Princeton University Press. See https://www.jstor.org/stable/j.ctt1kt82jg.

[4] Kokkoris GD, Troumbis AY, Lawton JH. 1999 Patterns of species interaction strength in assembled theoretical competition communities. Ecol. Lett. **2**, 70-74. (10.1046/j.1461-0248.1999.22058.x

[5] Maynard DS, Serván CA, Allesina S. 2018 Network spandrels reflect ecological assembly. Ecol. Lett. **21**, 324-334. (10.1111/ele.12912 29377488

[6] Chesson P. 2000 Mechanisms of maintenance of species diversity. Annu. Rev. Ecol. Syst. **31**, 343-366. (10.1146/annurev.ecolsys.31.1.343)

[7] Chase JM, Leibold MA. 2003 Ecological niches: linking classical and contemporary approaches. Chicago, IL: University of Chicago Press.

[8] Reynolds PL, Bruno JF. 2013 Multiple predator species alter prey behavior, population growth, and a trophic cascade in a model estuarine food web. Ecol. Monogr. **83**, 119-132. (10.1890/11-2284.1)

[9] Bairey E, Kelsic ED, Kishony R. 2016 High-order species interactions shape ecosystem diversity. Nat. Commun. **7**, 12285. (10.1038/ncomms12285) 27481625 PMC4974637

[10] Ratzke C, Barrere J, Gore J. 2020 Strength of species interactions determines biodiversity and stability in microbial communities. Nat. Ecol. Evol. **4**, 376-383. (10.1038/s41559-020-1099-4) 32042124

[11] May RM. 1972 Will a large complex system be stable? Nature **238**, 413-414. (10.1038/238413a0) 4559589

[12] Mougi A, Kondoh M. 2012 Diversity of interaction types and ecological community stability. Science **337**, 349-351. 22822151 10.1126/science.1220529

[13] Allesina S, Tang S. 2012 Stability criteria for complex ecosystems. Nature **483**, 205-208. (10.1038/nature10832) 22343894

[14] Wootton JT, Emmerson M. 2005 Measurement of interaction strength in nature. Annu. Rev. Ecol. Evol. Syst. **36**, 419-444. (10.1146/annurev.ecolsys.36.091704.175535)

[15] Aronson MFJ et al. 2014 A global analysis of the impacts of urbanization on bird and plant diversity reveals key anthropogenic drivers. Proc. R. Soc. B **281**, 20133330. (10.1098/rspb.2013.3330) PMC4027400 24523278

[16] Ushio M. 2019 Use of a filter cartridge combined with intra-cartridge bead-beating improves detection of microbial DNA from water samples. Methods Ecol. Evol. **10**, 1142-1156. (10.1111/2041-210X.13204)

[17] Ushio M, Murakami H, Masuda R, Sado T, Miya M, Sakurai S, Yamanaka H, Minamoto T, Kondoh M. 2018 Quantitative monitoring of multispecies fish environmental DNA using high-throughput sequencing. Metabarcoding Metagenomics **2**, e23297.

[18] Sugihara G, May R, Ye H, Hsieh C, Deyle E, Fogarty M, Munch S. 2012 Detecting causality in complex ecosystems. Science **338**, 496-500. (10.1126/science.1227079) 22997134

[19] Ye H, Beamish RJ, Glaser SM, Grant SCH, Hsieh CH, Richards LJ, Schnute JT, Sugihara G. 2015 Equation-free mechanistic ecosystem forecasting using empirical dynamic modeling. Proc. Natl Acad. Sci. USA **112**, E1569-E1576. (10.1073/pnas.1417063112) 25733874 PMC4386326

[20] Deyle ER, May RM, Munch SB, Sugihara G. 2016 Tracking and forecasting ecosystem interactions in real time. Proc. R. Soc. B **283**, 20152258. (10.1098/rspb.2015.2258) PMC4721089 26763700

[21] Callahan BJ, McMurdie PJ, Rosen MJ, Han AW, Johnson AJA, Holmes SP. 2016 DADA2: high-resolution sample inference from Illumina amplicon data. Nat. Meth. **13**, 581-583. 10.1038/nmeth.3869 PMC4927377 27214047

[22] Osada Y, Ushio M. 2021 rUIC: unified information-theoretic causality for R. Zenodo. (10.5281/zenodo.5163234)

[23] Cenci S, Sugihara G, Saavedra S. 2019 Regularized S-map for inference and forecasting with noisy ecological time series. Methods Ecol. Evol. **10**, 650-660. (10.1111/2041-210X.13150)

[24] Sugihara G. 1994 Nonlinear forecasting for the classification of natural time series. Phil. Trans. R. Soc. Lond. A **348**, 477-495. (10.1098/rsta.1994.0106)

[25] Chang CW, Miki T, Ushio M, Ke PJ, Lu HP, Shiah FK, Hsieh C. 2021 Reconstructing large interaction networks from empirical time series data. Ecol. Lett. **24**, 2763-2774. (10.1111/ele.13897) 34601794

[26] Ushio M, Hsieh C, Masuda R, Deyle ER, Ye H, Chang CW, Sugihara G, Kondoh M. 2018 Fluctuating interaction network and time-varying stability of a natural fish community. Nature **554**, 360-363. 29414940 10.1038/nature25504

[27] Cenci S, Saavedra S. 2019 Non-parametric estimation of the structural stability of non-equilibrium community dynamics. Nat. Ecol. Evol. **3**, 912-918. (10.1038/s41559-019-0879-1) 31036898

[28] Tilman D, Reich PB, Knops JMH. 2006 Biodiversity and ecosystem stability in a decade-long grassland experiment. Nature **441**, 629-632. 16738658 10.1038/nature04742

[29] Huston MA. 2014 Disturbance, productivity, and species diversity: empiricism vs. logic in ecological theory. Ecology **95**, 2382-2396. (10.1890/13-1397.1)

[30] Evans KL, Warren PH, Gaston KJ. 2005 Species-energy relationships at the macroecological scale: a review of the mechanisms. Biol. Rev. **80**, 1-25. (10.1017/S1464793104006517) 15727036

[31] Begon M, Townsend CR, Harper JL. 2005 Ecology: from individuals to ecosystems, 4th Edition. Hoboken, New Jersey: Wiley Blackwell Publishing. See https://www.wiley.com/en-us/Ecology%3A+From+Individuals+to+Ecosystems%2C+4th+Edition-p-9781405111171.

[32] Kasada M, Yamamichi M, Yoshida T. 2014 Form of an evolutionary tradeoff affects eco-evolutionary dynamics in a predator-prey system. Proc. Natl Acad. Sci. USA **111**, 16 035-16 040. (10.1073/pnas.1406357111) PMC4234545 25336757

[33] Tanabe AS, Toju H. 2013 Two new computational methods for universal DNA barcoding: a benchmark using barcode sequences of bacteria, archaea, animals, fungi, and land plants. PLoS ONE **8**, e76910. (10.1371/journal.pone.0076910) 24204702 PMC3799923

[34] R Core Team. 2019 R: a language and environment for statistical computing. Vienna, Austria: R Foundation for Statistical Computing.

[35] Takens F. 1981 Detecting strange attractors in turbulence. In Dynamical systems and turbulence (eds D Rand, L-S Young), pp. 366-381. New York, NY: Springer-Verlag. (10.1007/BFb0091924)

[36] Deyle ER, Sugihara G. 2011 Generalized theorems for nonlinear state space reconstruction. PLoS ONE **6**, e18295. (10.1371/journal.pone.0018295) 21483839 PMC3069082

[37] Ye H et al. 2018 rEDM: applications of empirical dynamic modeling from time series. (10.5281/zenodo.1294063)

